# Identification of risk factors for retinal vascular events in a population-based cross-sectional study in Rumoi, Japan

**DOI:** 10.1038/s41598-021-85655-y

**Published:** 2021-03-18

**Authors:** Reiko Kinouchi, Satoshi Ishiko, Kazuomi Hanada, Hiroki Hayashi, Daiki Mikami, Akitoshi Yoshida

**Affiliations:** 1 Medicine and Engineering Combined Research Institute, Asahikawa Medical University, Midorigaoka-Higashi 2-1-1-1, Asahikawa, 078-8510 Japan; 2 Department of Ophthalmology, Asahikawa Medical University, Asahikawa, Hokkaido Japan; 3 Asari Chuo Hospital, Hokkaido, Japan

**Keywords:** Diseases, Health care, Medical research

## Abstract

We conducted a population-based, cross-sectional study in Japan to identify risk factors for retinal vascular events separately by gender. Forty years or older participants were recruited. Fundus photographs were taken, and lifestyle and health characteristics were determined through a questionnaire and physical examinations. We compared the group of those who had retinal vascular events and those who did not. A total of 1689 participants (964 men) were deemed eligible for the study and retinal vascular events were seen in 59 subjects (3.7% of the men, 3.2% of the women). Self-reported diabetes mellitus was significantly associated with the vascular events in each gender [odds ratio (OR) = 6.97, 6.19 (men, women); 95% confidence interval (CI) 3.02–15.9, 2.25–17.0; p < 0.001]. Higher systolic blood pressure (OR = 1.03; 95% CI 1.01–1.04; p = 0.006) and lower frequency of meat consumption (OR = 0.73; 95% CI 0.54–0.99; p = 0.04) were independently associated with the vascular events in men. In women, while vascular events were associated with self-reported hypertension (OR = 2.64; 95% CI 1.03–6.74; p = 0.04), no association was seen with systolic blood pressure. Women with hypertension may need extra care, not only for blood pressure.

## Introduction

The retinal vasculature can be observed using fundus photographs and it is one of the easiest regions in the body to observe the microvasculature condition. Retinal vascular events, including retinal hemorrhages, microaneurysms, cotton-wool spots and hard exudates can be predictive indicators for stroke and cardiovascular events^1–3^. Knowing risk factors for retinal vascular events, and avoiding modifiable risk factors, can contribute to reducing the risk of systemic vascular events.

There have been several population-based studies analyzing risk factors for retinal vascular events among individuals with and without diabetes. Higher systolic blood pressure^4–7^, chronic kidney disease^4,6,7^, older age^4^ and metabolic syndrome^8^ have been reported as risk factors for retinal vascular events. Few reports have addressed modifiable risk factors, such as lifestyle factors. To determine modifiable risk factors for retinal vascular events, we conducted a population-based, cross-sectional study in Rumoi, in northern Japan. Because blood pressure and lifestyle vary by gender^9^, and gender differences in risk factors for retinal vascular events have also been suggested^10^, we analyzed risk factors separately by gender.

## Materials and methods

### Study design and participants

Our population-based, cross-sectional study in Rumoi was described in detail previously^9^. Briefly, we recruited 1731 participants (11% of the residents of Rumoi city in Japan) aged 40 years old or older, from January 2013 to March 2015. The study was carried out in accordance with the tenets of the Declaration of Helsinki and it was approved by the research ethics committee of Asahikawa Medical University.

### Procedures

As we described detail previously^9^, after obtaining written informed consent, we checked the participants’ prescription records and asked them to complete a questionnaire detailing their occupation, lifestyle habits and health information, including any present illness. They also underwent a physical examination that included measurement of height, body weight, systolic and diastolic blood pressure, pulse rate and body composition; intraocular pressure was also measured, using the NT-5P non-contact tonometer (Nidek, Aichi, Japan). One non-stereoscopic, color 45-degree fundus photograph of each eye, centered on the macula, was taken using an AFC-230 non-mydriatic auto fundus camera, which has a resolution of 21.1 million pixels (Nidek, Aichi, Japan).

We defined retinal vascular events as the presence of at least one of following findings: retinal hemorrhage, retinal vascular occlusions, optic disc hemorrhage, cotton wool spots and hard exudates. We did not distinguish retinal microaneurysms from retinal dot hemorrhages, and all of those were classified as retinal hemorrhages.

**Figure 1:**
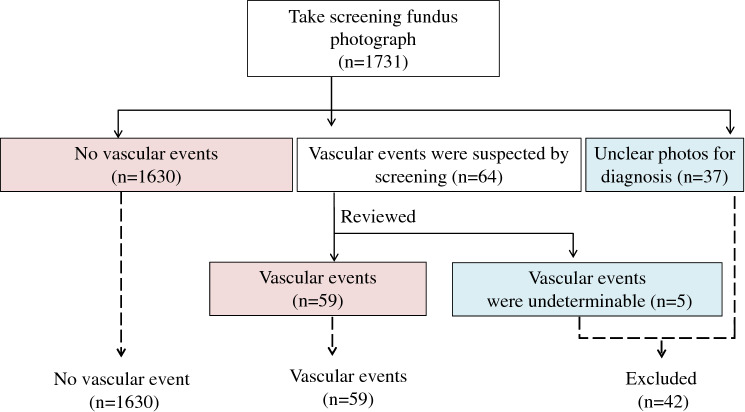
Summary of the participant groups included in the study. Among the 1731 participants, 37 were excluded because of unclear photographs that could not be used to diagnose pathological findings requiring further investigation. Suspected findings of retinal vascular events were identified in fundus photographs of 64 participants by the initial screening. Further reviews of the participants’ photographs led to the exclusion of five participants because their findings were indistinguishable from changes other than vascular events such as pigmentations. Finally, we included 1630 in the no-retinal vascular event group and 59 in the retinal vascular event group. We excluded 42 participants who either had unclear fundus photographs or who could not be definitively diagnosed as having a retinal vascular event.

The fundus photographs were not taken specifically for this study; they were part of examinations to detect pathological findings requiring further investigation, as described in a previous study^9^. The fundus photographs of 1731 participants were initially screened by ophthalmology specialists (authors RK, SI, or KH) for any pathological findings requiring further investigation; 37 participants were excluded because their photographs were insufficient for diagnosis. We extracted retinal vascular events and suspected findings among 1694 participants’ fundus photographs, and author RK reviewed them (Fig. 1).

### Statistical analysis

The crude prevalence of retinal vascular events was calculated, and age-adjusted standardized prevalence was calculated using the direct method of the ‘epitools’ package, version 0.5–10.1 with R version 3.6.3 (The R Foundation for Statistical Computing Platform, Vienna, Austria) with reference to the age distribution of the entire Rumoi population, which was based on the information contained in the basic resident register on January 2015^9^. We statistically compared the participants who had retinal vascular events and those who did not. As the physiological data and lifestyles differed by gender (Supplementary tables S1, S2), we analyzed the results from men and women separately. Fisher’s exact test and Wilcoxon rank-sum test were used for univariate analyses, and logistic regression was performed for multivariate analyses. We used analysis of variance (ANOVA) to adjust for age between groups. For logistic regression, we entered as variables the factors that had significant association with the retinal vascular events in the univariate analysis in the current study. For these analyses, R version 3.6.3 and EZR (Jichi Medical University, Saitama, Japan), a graphical user interface for R, were used^11^. We included disc hemorrhage, which is one of the risk factors for glaucoma, as a vascular event. Thus, we compared the participants who had disc hemorrhage and those who had the other types of retinal vascular events, and also compared intraocular pressures of those with disc hemorrhage with those who had no vascular events, for reference.

### Ethics approval

The current study was approved by the research ethics committee of Asahikawa Medical University.

## Results

### Characteristics of subjects

Retinal vascular events, or suspected findings, were seen in 64 participants’ photographs. After reviewing the photographs, five participants were excluded as their photographs were indistinguishable from other changes such as image artifacts and pigmentation (Fig. 1). A total of 1689 participants (964 men and 725 women) were deemed eligible for the study cohort. The age range of the cohort was 40–89 years, and the mean age [mean ± standard deviation (SD)] was 56.0 ± 10.6 years in men and 60.2 ± 11.0 years in women. The age distributions are shown in Supplementary table S3.

### Prevalence and details of retinal vascular events

Retinal vascular events were observed at least unilaterally in 59 participants. The crude prevalence of retinal vascular events in participants 40 years old or older was 3.5%, and the age-adjusted prevalence was 4.2% [95% confidence interval (CI) 2.9–6.3%]. The crude prevalence in those participants 50 years old or older was 4.2%, and the age-adjusted prevalence was 4.9% (3.3–7.4%) (Table 1, Supplementary table S3).

**Table 1:** Prevalence of retinal vascular events.

	Crude%	Age-adjusted%		(95% CI)
**40 years and older**				
Men	3.7		4.7	(2.9–7.8)
Women	3.2		3.5	(1.9–6.9)
Men and women	3.5		4.2	(2.9–6.3)
**50 years and older**				
Men	4.7		5.6	(3.3–9.5)
Women	3.7		4.0	(2.1–8.1)
Men and women	4.2		4.9	(3.3–7.4)

*CI* confidence interval.

Details of the vascular events in the 59 participants are shown in Table 2. The retinal vascular events were unilateral in 39 participants and bilateral in 20. Among the retinal vascular events, six participants had, or were suspected to have, a retinal vein occlusion (RVO), and six had, or were suspected to have, diabetic retinopathy. We suspected RVO when a few retinal hemorrhages were observed near small sites of arteriovenous nicking, or when white occlusive vessels suggestive of old RVO were observed. We suspected diabetic retinopathy when several dot retinal hemorrhages and exudates were observed.

**Table 2:** Details of the retinal vascular events.

	Unilateral	Bilateral	total
	n	n	n
**Retinal hemorrhage**	30	15	45*
Hemorrhage only	29	10	39
With vascular occlusion	1	1	2
With hard exudate	0	1	1
With cotton wool spot	0	2	2
With abnormal vessels	0	1	1
**Disk hemorrhage**	6	1	7
**Hard exudate**	2	0	2
**Cotton wool spot**	1	0	1
**Retinal vascular occlusion**	0	4	4
Total	39	20	59

*Four of them had suspected retinal arteriolar macroaneurysms.

### Statistical comparison of those who had retinal vascular events and those who did not

Univariate comparisons of the participants who had retinal vascular events and those who did not are shown in Table 3. Self-reported diabetes mellitus (p < 0.001) and older age (men; p = 0.002, women; p = 0.02) were associated with the vascular events in both genders, and self-reported hypertension was associated with the events in the women (p < 0.001) but not in the men (p = 0.83). Smoking (p = 0.02), higher systolic blood pressure (p = 0.01), lower frequency of consumption of meat (p = 0.006) and coffee (p = 0.01) were associated with the vascular events in the men but not in the women. Further using ANCOVA to adjust for age, and logistic regression analysis to adjust for age and self-report diabetes, systolic blood pressure didn’t show association with the vascular events in the women (p = 0.26, 0.24; Supplementary figure S4, table S5).

**Table 3:** Results of univariate comparisons of the participants who had retinal vascular events and those who did not.

Parameter	Men			Women		
	Vascular event	No vascular event	*P V*alue	Vascular event	No vascular event	*P V*alue
	(n = 36)	(n = 928)		(n = 23)	(n = 702)	
Gender, men; n (%)	36 (61)	928 (57)				0.59^#^
Age (years.); mean (SD)	61.2 (10.0)	55.8 (10.5)	0.002**	65.4 (9.0)	60.0 (11.0)	0.02*
**Questionnaire**						
Self-report of diabetes; n (%)	11 (31)	59 (6)	^#^ < 0.0001**	7 (30)	32 (5)	^#^ < 0.0001**
Self-report of hypertension; n (%)	6 (17)	182 (20)	^#^0.83	12 (52)	143 (20)	^#^0.0009**
Have occupation; n (%)	30 (83)	778 (84)	^#^1.00	7 (30)	350 (50)	^#^0.09
Blood type (A-B-O-AB); n (%)	18–7-7–4 (50–19-19–11)	337–203-292–96 (36–22-31–10)	^#^0.31	10–8-5–0 (43–35-22–0)	253–146-227–76 (36–21-32–11)	^#^0.12
Number of family living together (include oneself); mean (SD)	2.4 (1.1)	2.6 (1.2)	0.26	2.0 (0.9)	2.4 (1.1)	0.17
**Activity; mean (SD)**						
Walking (hours/day)	1.8 (1.5)	2.0 (2.2)	0.67	2.7 (2.0)	2.9 (2.7)	0.98
Exercise (hours/week)	2.5 (5.5)	2.0 (3.7)	0.67	2.3 (3.8)	2.0 (4.4)	0.54
**Habits; mean (SD)**						
Smoking (number × years)	637 (521)	443 (416)	0.02*	61 (136)	95 (219)	0.38
Coffee (cups/day)	1.7 (2.7)	2.1 (1.7)	0.01*	1.8 (1.3)	1.8 (1.4)	0.75
Tea (cups/day)	1.8 (2.2)	1.2 (1.6)	0.13	2.6 (2.4)	1.8 (2.1)	0.10
Alcohol (glasses/day)	1.4 (1.5)	1.2 (1.6)	0.43	0.1 (0.3)	0.4 (0.9)	0.25
Fruit (number/day)	0.6 (0.7)	0.6 (0.7)	0.63	1.0 (0.7)	0.9 (0.7)	0.44
Meat (eating days/week)	1.9 (1.1)	2.5 (1.4)	0.006**	2.3 (1.9)	2.6 (1.5)	0.31
Fish (eating days/week)	3.5 (1.7)	2.9 (1.6)	0.06	3.8 (2.1)	3.5 (1.6)	0.55
**Measurements; mean (SD)**						
Body mass index (kg/m^2^)	25.2 (4.0)	24.8 (3.6)	0.97	24.7 (4.3)	23.3 (3.9)	0.12
Systolic blood pressure(mmHg/Ag)	142 (21)	134 (19)	0.01*	134 (18)	127 (19)	0.07
Diastolic pressure(mmHg/Ag)	81.6 (14.1)	79.5 (11.7)	0.76	77.5 (12.6)	76.7 (11.0)	0.64
Pulse rate/minute	79.7 (17.0)	77.1 (12.5)	0.40	76.0 (11.6)	76.8 (11.8)	0.73
**Estimated values**						
basal metabolism (kcal/day)	1487 (174)	1505 (196)	0.50	1089 (123)	1096 (121)	0.77
Muscle (kg)	51.5 (5.1)	52.0 (5.9)	0.54	35.3 (3.2)	35.5 (3.2)	0.54
Bone mass (kg)	2.8 (0.3)	2.8 (0.3)	0.53	2.1 (0.3)	2.1 (0.3)	0.45
Body fat (%)	16.5 (7.6)	15.2 (7.6)	0.31	19.1 (7.0)	17.6 (7.6)	0.22
Body water (%)	38.3 (4.7)	38.0 (5.0)	0.84	27.9 (3.5)	27.7 (3.1)	0.68
**Intraocular pressure (mmHg/Ag)**						
Left eye	14.5 (2.4)	14.0 (2.8)	0.25	14.5 (2.9)	14.1 (2.8)	0.37
Right eye	14.8 (3.0)	14.2 (2.7)	0.28	13.8 (2.3)	14.2 (2.8)	0.65

*SD* standard deviation * < 0.05; ** < 0.01; ^#^P Value calculated by Fisher's exact test.; P value without # is calculated by Wilcoxon rank sum test.

The results of the multivariate logistic regression analysis, using as variables the factors that had significant association with retinal vascular events in the univariate analysis, are shown in Table 4. Self-reported diabetes mellitus was significantly associated with the vascular events in both genders (men; OR = 6.92; 95% CI 3.02–15.9; p < 0.001, women; OR = 6.19; 95% CI 2.25–17.0; p < 0.001). Higher systolic blood pressure (OR = 1.03; 95% CI 1.01–1.04; p = 0.006) and lower frequency of meat consumption (number of days/week, OR = 0.73; 95% CI 0.54–0.99; p = 0.04) were associated with the vascular events in men, and self-reported hypertension was independently associated with the vascular events in women (OR = 2.64; 95% CI 1.03–6.75; p = 0.04).

**Table 4:** Multivariate risk factors associated with retinal vascular events.

Parameter	Odds ratio (95% confidence interval)		*P V*alue
**Men**			
Age	1.02	(0.99–1.06)	0.25
Self-report of diabetes	6.92	(3.02–15.9)	< 0.001**
Systolic blood pressure(mmHg/Ag)	1.03	(1.01–1.04)	0.006**
Smoking (number × years)	1.00	(1.00–1.00)	0.15
Coffee (cups/day)	0.93	(0.75–1.17)	0.54
Meat (eating days/week)	0.73	(0.54–0.99)	0.04*
**Women**			
Age	1.02	(0.97–1.06)	0.50
Self-report of diabetes	6.19	(2.25–17.0)	< 0.001**
Self-report of hypertension	2.64	(1.03–6.74)	0.04*
Systolic blood pressure(mmHg/Ag)	1.01	(0.99–1.04)	0.33

* < 0.05, ** < 0.01.

### Analysis of disc hemorrhage

Disc hemorrhages were observed in seven participants. Six of them took detailing examination at local clinics; one was diagnosed with open angle-glaucoma, and five were not. By statistical analysis, there were no significant differences in univariate comparisons of the factors between the participants who had disc hemorrhage and those with the other retinal vascular events (Supplementary table S6). No significant difference was seen in intraocular pressure between the participants who had disc hemorrhage and the participants who had no retinal vascular event (Supplementary table S7).

## Discussion

We assessed risk factors for retinal vascular events using the data of the Rumoi study, a population-based, cross-sectional study in Japanese individuals, aged 40 or older. The age-adjusted prevalence of retinal vascular events was 4.2% (95% CI 2.9–6.3%) in those 40 years old or older, and 4.9% (95% CI 3.3–7.4%) in those 50 years old or older, in the current study. Self-reported diabetes mellitus in each gender, higher systolic blood pressure and lower frequency of meat consumption in men, and self-reported hypertension in women were each independently associated with retinal vascular events.

In the current study, we defined retinal vascular events as the presence of at least one of following findings: retinal hemorrhages/retinal microaneurysms, disc hemorrhage, hard exudates, cotton wool spots and retinal vascular occlusions. Diabetic retinopathy and RVO were included among the retinal vascular events, as the diseases had these findings. In the Third National Health and Nutrition Examination Survey (NHANES III) and the Beaver Dam Eye Study, when at least one of finding of retinal microaneurysms, hemorrhages, soft exudates, hard exudates, intraretinal microvascular abnormalities or diabetic retinopathy was seen, the authors defined them as retinopathies^12,13^. Although disc hemorrhage and retinal vascular occlusion were not specifically identified in the earlier articles, those may be included in the definition of retinopathy, as they could be considered retinal hemorrhages. In that case, the earlier definition of retinopathy and our definition of retinal vascular events are similar.

Disc hemorrhage is known to be a risk factor for glaucoma^14^, and the etiology of disc hemorrhage is controversial whether it is caused by vascular factors or intraocular pressure. We compared intraocular pressure between subjects who had disc hemorrhage and those who had no retinal vascular event (Supplementary table S7). There was no significant difference in intraocular pressure between the two groups. We also compared the participants who had disc hemorrhage and those who had other vascular events. There was no significant difference between the groups in any of the variables (Supplementary table S6). Therefore, we considered that disc hemorrhage could be included in one of the retinal vascular events.

In the NHANES III population, 5.4% of those 40 years old or older had retinopathy^12^, 7.3% of the nondiabetic persons in the Beaver Dam Eye Study cohort had retinopathy^13^, and 13% of those 50 years old or older in a Beijing study had fundus hemorrhage^5^. In the current study, the age-adjusted prevalence of vascular events was 4.9% (95% CI 3.3–7.4%) in the 50-years-and-older cohort, and 4.2% (95% CI 2.9–6.3%) in the 40-years-and-older cohort. Thus, the prevalence of retinal vascular events was similar to that in the NHANES III sample, but lower than those in the Beaver Dam Eye Study and the Beijing study. This may be caused by the different methods used to detect fundus findings between the studies. NHANES III and the current study each evaluated a single photograph of each eye, whereas the Beijing study and Beaver Dam Eye Study used multiple fundus photographs of each eye for their evaluations. Even with a single fundus photograph for each eye, taken of the posterior pole, around 5% of the 40-years-and-older population had retinal vascular events or retinopathy in this cross-sectional, population-based study. That means that retinal vascular events are not rare findings and that it is useful to analyze the background factors related to microvascular changes.

While higher systolic blood pressure was an independent risk factor for the retinal vascular events for men in the current study, self-reported hypertension was not related to retinal vascular events in the men. The result suggests that controlling blood pressure can reduce the risk of retinal vascular events, even for men who are diagnosed with hypertension or diabetes mellitus. This supports results reported previously^4–6,15^ Conversely, self-reported hypertension, but not blood pressure, was independently associated with the retinal vascular events for women in the current study. Blood pressure was not associated with retinal vascular events in women either using the univariate analysis or age adjusted analysis (p = 0.26, Supplementary figure S4). This result suggests the retinal vessels in women with hypertension are already vulnerable. Differences in blood pressure by gender are known; blood pressure in young women is lower than in young men, and postmenopausal blood pressure elevation is seen in women^16,17^. Women who have hypertension may need additional care to keep their vessels durable, and may need earlier diagnosis to start treatment before the vessels become vulnerable.

In the Tromsø Eye Study^10^, the authors investigated gender-stratified risk factors for retinopathy without diabetes. They showed a strong association between blood pressure and retinopathy in both genders, but a significant association between retinopathy and microalbuminuria was only present in women. Consistent with the prior report, gender differences in risk factors were observed in the current study, but the types of differences were distinct. In the current study, higher systolic blood pressure was a risk factor for retinal vascular events in men, but not in women. This difference in risk factors between studies is presumably related to differences in subject populations: subjects in the current study were Asian, rather than white/Caucasian, and included patients with diabetes. The mean systolic blood pressure was lower among subjects in the current study than among subjects in the Tromsø Eye Study, especially in women (approximately 10 mmHg lower). Because we did not examine microalbuminuria, we could not assess that risk factor. Although our findings concerning gender differences in types of risk factors differed from those in the Tromsø Eye Study, our results support their speculation that gender differences can be an underlying cause of retinopathy^10^.

We also explored lifestyle risk factors for retinal vascular events. A lower frequency of eating meat was independently associated with retinal vascular events in men (p = 0.04). Our classification of meat included pork, beef, chicken and meat from other birds or animals in the current study; pork is the most common meat to be consumed in Hokkaido prefecture where we conducted the study. Although there have been several reports regarding the relation of vascular diseases and red meat^18–22^, we could not find articles indicated the relation of the retinal vascular events and meat. Japanese meat consumption is lower than that in Western countries^23^, and in the current study, the mean frequency of meat consumption in men was 1.9 days/week in the retinal vascular event group and 2.5 days/week in the no-retinal vascular event group. The result suggests that a proper frequency of meat consumption is needed to keep retinal vasculature healthy.

Higher age in both genders, and more smoking and lower consumption of coffee in men were associated with the retinal vascular events by univariate comparison, but not by multivariate comparison in the current study. Higher age and smoking were positively associated with self-reported diabetes (Supplementary table and figures S8), and that may affect the results of the univariate comparison, according to which, higher age and smoking were positively associated with retinal vascular events in men. Although there was no significant relation of consumption of coffee with diabetes or blood pressure, older individuals tended to consume less coffee, and that may influence the results (Supplementary table and figure S9).

This study has some limitations. First, although we recruited 11% of the residents of Rumoi city who were aged 40 years or older and the prevalence of retinal vascular events did not considerably differ from the findings in previous studies, the participants were not randomly selected. Because the participants needed to visit us for a screening examination, those who were in poor health or who had poor visual function could not be included. In addition, because this study only included a small number of subjects with retinal vascular events, biased subject selection might have affected the results. Second, only a single fundus photograph was taken of each eye for evaluation. Thus, the prevalence of retinal vascular events in the current study may be lower than the actual prevalence of retinal vascular events. Third, diabetic mellitus and hypertension were self-reported. We checked prescription records and tried to not miss any diagnoses of diabetes or hypertension, but it is possible that not all cases were detected. Fourth, the items on the questionnaire we used regarding lifestyle and eating habits were limited, and so there might be missing confounding factors related to retinal vascular events.

In conclusion, we conducted a cross-sectional study of the Japanese population, the age-adjusted prevalence of retinal vascular events was 4.2% (95% CI 2.9–6.3%) in the 40-years-and-older cohort. Diabetic mellitus was strongly associated with retinal vascular events in both genders. While systolic blood pressure appears to be an independent risk factor for retinal vascular events in men, a diagnosis of hypertension does not. Thus, effective blood pressure control could lower the risk of retinal vascular events in men. Hypertension appears to be a risk factor for retinal vascular events in women, but systolic blood pressure does not. Women with hypertension may need extra care, not only for blood pressure, and may need earlier diagnosis to start treatment earlier. Some lifestyle risk factors for the vascular events, such as lower meat consumption, were suggested in men.

## Supplementary information

Supplementary information.

## Data Availability

The datasets generated and analyzed during the current study are available from the corresponding author on reasonable request.
