# Supplementary material for: Identification of risk factors for retinal vascular events in a population-based cross-sectional study in Rumoi, Japan
Source: Sci Rep. 2021 Mar 18;11:6340. doi: 10.1038/s41598-021-85655-y (PMC7973805; doi:10.1038/s41598-021-85655-y)

Identification of risk factors for retinal vascular events in a population-based cross-sectional study in Rumoi, Japan

Reiko Kinouchi, [1,2,*] Satoshi Ishiko,[1,2] Kazuomi Hanada,[1,3] Hiroki Hayashi,[1] Daiki Mikami,[1] Akitoshi Yoshida,[2]

Supplementary Information

Supplementary Table S1. Results of univariate comparisons of men and women in the current study

Parameter	men (n =968)	women (n =726)	*P V*alue
Age (yrs); mean (SD)	56 (11)	60 (11)	<0.001 **
Questionnaire			
Self-report diabetes; n (%)	73 (8)	39 (5)	# 0.08
Self-report hypertension; n (%)	189 (20)	156 (21)	# 0.33
Have occupation; n (%)	809 (84)	357 (49)	# <0.001 **
blood type (A-B-O-AB) ; n (%)	358-211-299-100 (37-22-31-10)	263-154-232-77 (36-21--32-11)	# 0.96
Number of family living together (include oneself) ; mean (SD)	2.6 (1.2)	2.4 (1.1)	<0.001 **
Activity; mean (SD)		( )	
Walking （hours/day）	2.0 (2.2)	2.9 (2.6)	<0.001 **
Exercise（hours/week）	2.0 (3.7)	2.0 (4.3)	0.34
Habits; mean (SD)	( )	( )	
Smoking （number x years ）	23 (21)	5 (11)	<0.001 **
Coffee （cups/day）	2.1 (1.7)	1.8 (1.4)	0.02 *
Tea (cups/day)	1.2 (1.7)	1.8 (2.2)	<0.001 **
Alcohol （glasses/day）	1.2 (1.6)	0.4 (0.9)	<0.001 **
Fruit (number/day)	0.6 (0.7)	0.9 (0.7)	<0.001 **
Meat (eating days/week)	2.5 (1.4)	2.6 (1.5)	0.004 **
Fish (eating days/week)	2.9 (1.6)	3.5 (1.7)	<0.001
Measurments; mean (SD)			
Body mass index (kg/m^2)	25 (4)	23 (4)	<0.001 **
Systolic blood pressure(mmHg/Ag)	134 (19)	127 (19)	<0.001 **
Diastolic pressure(mmHg/Ag)	80 (12)	77 (11)	<0.001 **
Pulse rate/minute	77 (13)	77 (12)	0.64
Estimated values			
basal metabolism (kcal/day）	1504 (196)	1095 (121)	<0.001 **
Muscle (kg)	52 (6)	36 (3)	<0.001 **
Bone mass (kg)	2.8 (0.3)	2.1 (0.3)	<0.001 **
Body fat (%)	15 (8)	18 (8)	<0.001 **
Body water (%)	38 (5)	28 (3)	<0.001 **
Intraocular pressure (mmHg/Ag)			
left eye	14.1 (2.8)	14.1 (2.8)	0.84
right eye	14.2 (2.8)	14.2 (2.8)	0.82

SD = standard deviation * < 0.05 **< 0.01

#: *P* value calculated by Fisher's exact test.; *P* value without # is calculated by Wilcoxon rank sum test.

Supplementary Table S2. Multivariate factor associations for men compared with women in the current study

Parameter	Odds Ratio (95% Confidence Interval)	*P Value*	
Age	1.01 (1.00 - 1.03)	0.20	
Have occupation	4.18 (2.99 - 5.85)	<0.001	**
Number of family living together (include oneself)	1.08 (0.97 - 1.22)	0.17	
Walking （hours/day）	0.83 (0.78 - 0.87)	<0.001	**
Smoking （pack x years ）	1.07 (1.06 - 1.08)	<0.001	**
Coffee （cups/day）	0.98 (0.91 - 1.07)	0.68	
Tea (cups/day)	0.99 (0.93 - 1.06)	0.83	
Alcohol（glasses/day）	1.66 (1.46 - 1.89)	<0.001	**
Fruit (number/day)	0.86 (0.70 - 1.04)	0.13	
Meat (eating days/week)	0.91 (0.83 - 1.00)	0.04	*
Fish (eating days/week)	0.86 (0.79 - 0.94)	<0.001	**

multivariate logistic regression analysis

Supplementary Table S3. Age distribution of all subjects and of the subjects in the retinal vascular event group

Age groups	Men (n=964)		Women (n=725)	
years	Participants; n	Vascular events; n (%)	Participants; n	Vascular events; n (%)
40-49	306	5 (1.6)	152	2 (1.3)
50-59	307	12 (3.9)	175	2 (1.1)
60-69	240	12 (5.0)	239	11 (4.6)
70-79	92	5 (5.4)	137	7 (5.1)
80-89	19	2 (10.5)	22	1 (4.5)
Total 40 and older	964	36 (3.7)	725	23 (3.2)
Total 50 and older	658	31 (4.7)	573	21 (3.7)

Supplementary Figure S4. Comparison of systolic blood pressure between participants who had retinal vascular events and those who had no retinal vascular event in women.

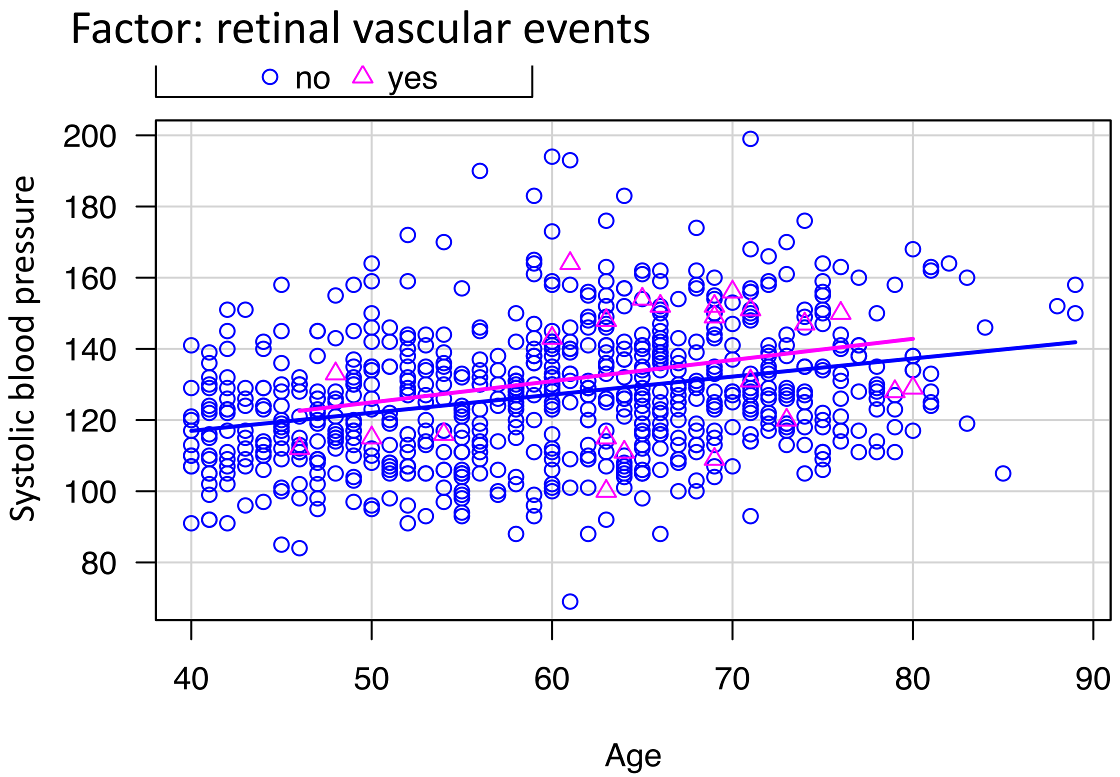

Scatter plot and regression line of age versus systolic blood pressure in women who had retinal vascular events and who didn't. No association was seen between retinal vascular events and age adjusted systolic blood pressure in women (*p*=0.26 analyzed using ANCOVA) .

Supplementary Table S5 . Risk factors associated with retinal vascular events; adjusted model

Parameter	Odds Ratio (95% Confidence Interval)		P Value
Men[a]			
Smoking（number x years）	1.01	(1.00 - 1.02)	0.17 [a]
Coffee (cups/day)	0.95	(0.77 - 1.18)	0.63 [a]
Meat (eating days/week)	0.74	(0.54 - 1.00)	0.05 [a*]
Women[b]			
Self-report hypertension	2.80	(1.10 - 7.14)	0.03 [b*]
Systolic blood pressure(mmHg/Ag)	1.01	(0.99 - 1.04)	0.24 [b]

[a]Adjusted for age, self-report diabetes and systolic blood pressure using logistic regression analysis.

[b]Adjusted for age and self-report diabetes using logistic regression analysis.

* < 0.05

Supplementary Table S6. Univariate comparison between participants who had disc hemorrhage and those who had the other retinal vascular events

Parameter	Disc hemorrhage (n =7)		the other vasucular events (n =52)			*P Value*
Age (yrs) ; mean (SD)	69.0	(7.5)	62.0	(9.8)		0.06
Gender; n (%) male-femail	4-3	(57-43)	32-20	(62-38)	#	1.00
Questionnaire						
Diabetes mellitus; n (%)	2	(29)	16	(31)	#	1.00
Hypertension; n (%)	3	(43)	15	(29)	#	0.66
Have occupation; n (%)	2	(29)	35	(67)	#	0.09
blood type (A-B-O-AB) ; n (%)	3-3-1-0	(43-43-14-0)	25-12-11-4	(48-23-21-8)	#	0.80
Number of family living together (include oneself) ; mean (SD)	2.7	(1.2)	2.2	(1.0)		0.41
Activity; mean (SD)						
Walking （hours/day）	2.1	(2.3)	2.1	(1.7)		0.60
Exercise（hours/week）	3.3	(5.0)	2.3	(4.9)		0.46
Habits; mean (SD)						
Smoking （number x years ）	24	(36)	21	(24)		0.79
Coffee （cups/day）	1.6	(1.5)	1.8	(2.3)		0.95
Tea (cups/day)	2.7	(2.0)	2.0	(2.3)		0.29
Alcohol （glasses/day）	0.9	(1.7)	0.9	(1.3)		0.54
Fruit (number/day)	1.3	(0.7)	0.7	(0.7)		0.07
Meat (eating days/week)	2.0	(0.8)	2.1	(1.5)		0.84
Fish (eating days/week)	4.3	(2.1)	3.5	(1.8)		0.23
Measurments; mean (SD)						
Body mass index (kg/m^2)	26	(5)	25	(4)		0.84
Systolic blood pressure(mmHg/Ag)	134	(18)	140	(20)		0.49
Diastolic pressure(mmHg/Ag)	80	(11)	80	(14)		0.92
Pulse rate/minute	75	(14)	79	(15)		0.53
Estimated values						
basal metabolism (kcal/day）	1281	(284)	1339	(244)		0.67
Muscle (kg)	44	(9.9)	45.4	(8.9)		0.74
Bone mass (kg)	2.4	(0.5)	2.5	(0.4)		0.56
Body fat (%)	19	(8.8)	17.3	(7.3)		0.73
Body water (%)	35	(6.5)	34.2	(6.6)		0.91
Intraocular pressure (mmHg/Ag)						
left eye	13.6	(3.5)	14.6	(2.4)		0.39
right eye	14.5	(3.7)	14.4	(2.6)		0.74

#: *P* value calculated by Fisher's exact test.; *P* value without # is calculated by Wilcoxon rank sum test.

Supplementary Table S7. Comparison of intraocular pressures between participants who had disc hemorrhage and those who had no retinal vascular event.

Parameter	Disc hemorrhage (n =7)	no vasucular events (n =1630)	*P V*alue	
Age; mean (SD) yrs	69 (7.5)	58 (11)	0.01	#1
Gender; n (%) male-femail	4-3 (57-43)	928-702 (57-43)	1.00	#2
Intraocular pressure; mean (SD) mmHg/Ag				
left eye	13.6 (3.5)	14.0 (2.8)	0.63	#3
right eye	14.5 (3.7)	14.2 (2.8)	0.78	#3

#1: *P* value calculated by Wilcoxon rank sum test with continuity correction

#2: *P* value calculated by Fisher exact test

#3: *P* value adjusted by age using ANCOVA

Since disc hemorrhages were observed more in higher age participants (#1), we compared intraocular pressure between participants who had disc hemorrhage and those who had no retinal vascular using ANCOVA to adjust age. No difference in intraocular pressures were seen between participants who had disc hemorrhage and those who had no retinal vascular event (#3).

Supplementary Table and Figures S8. Multivariate risk factors associated with self-report of diabetes mellitus and box plot for diabetes versus smoking and coffee drinking in men

Multivariate risk factors associated with self-report of diabetes mellitus in men

Parameter	Odds Ratio (95% Confiedece Interval)	*P Value*
Age	1.03 (1.01 - 1.06)	0.006
Coffee (cups/day)	1.07 (0.94 - 1.22)	0.32
Smoking (number x years)	1.00 (1.00 - 1.00)	0.001

multivariate logistic regression analysis

Box plot for diabetic by smoking in men

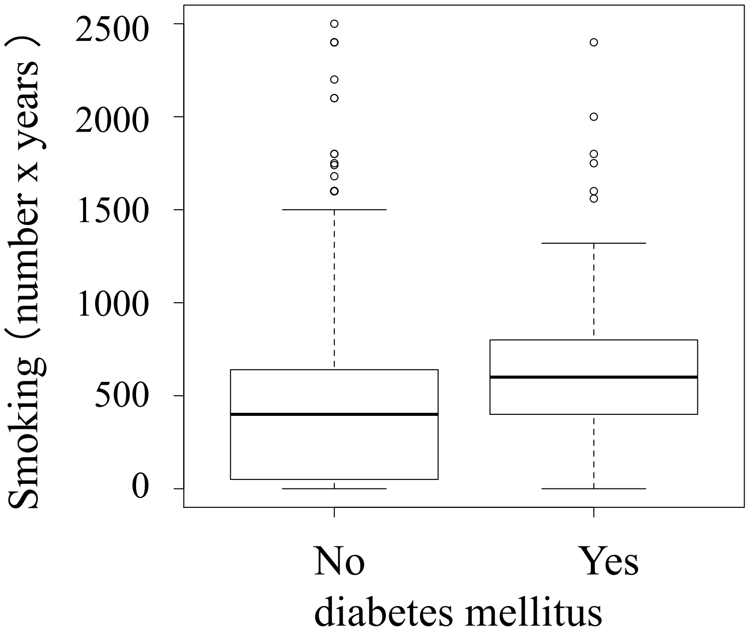

Box plot for diabetic by coffee drinking in men

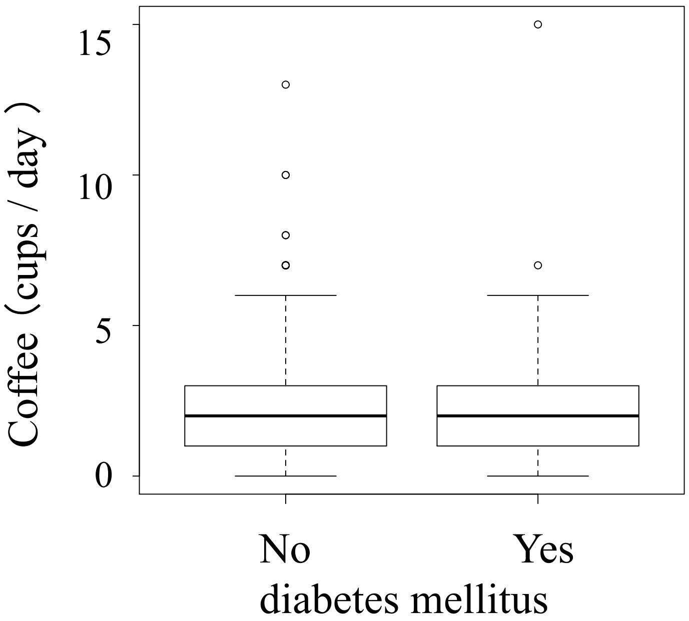

Age and smoking were associated with self-report of diabetes mellitus in men, but coffee drinking was not associated with self-report of diabetes mellitus .

Supplementary Table and figures S9. Correlation between coffee drinking and age, smoking, or systolic blood pressure in men based on the Pearson correlation coefficient.

Correlation between coffee drinking	Coefficient	Confidence interval	*P* Value
Age	-0.17	-0.24--0.12	<0.0001
Smoking	0.04	-0.02-0.10	0.21
Systolic blood pressure	-0.06	-0.12-0.0026	0.06

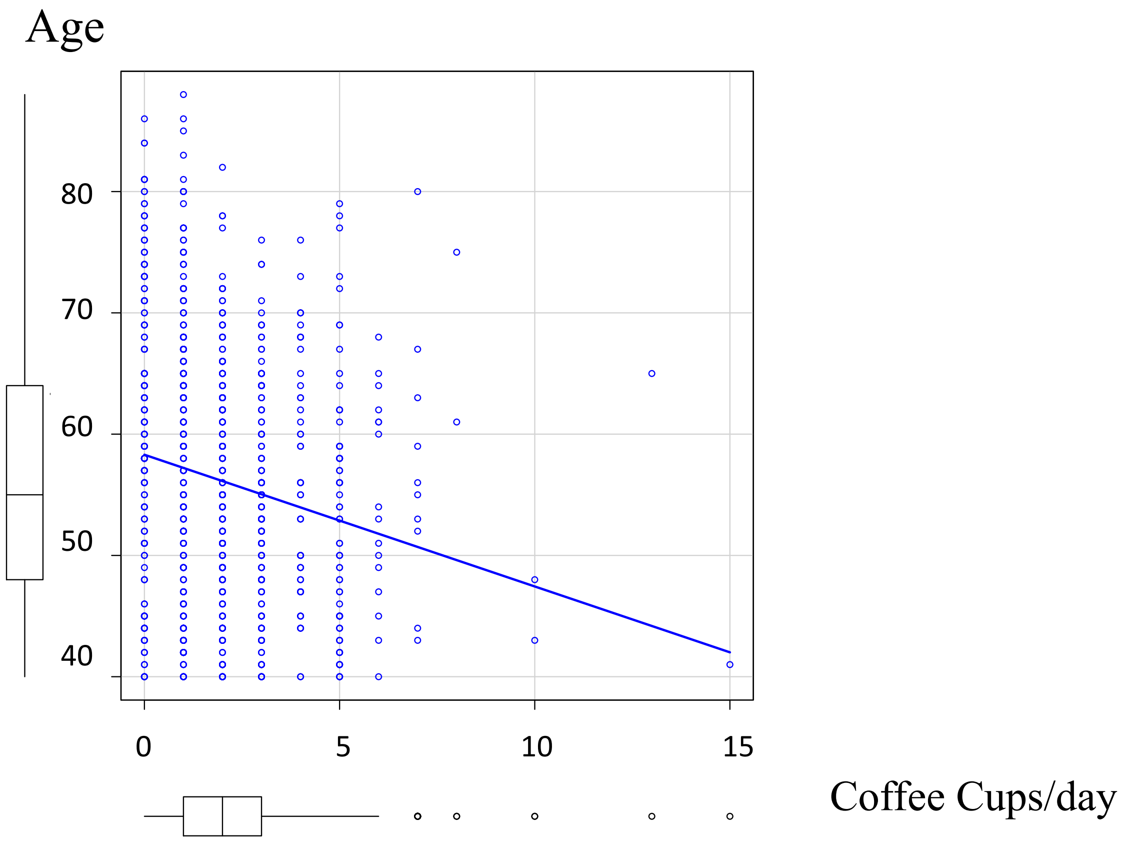

Supplement: Supplementary file 1 — Supplementary information. [file 41598_2021_85655_MOESM1_ESM.pdf]
